# Effects of Aerobic Exercise on Cortisol Stress Reactivity in Response to the Trier Social Stress Test in Inpatients with Major Depressive Disorders: A Randomized Controlled Trial

**DOI:** 10.3390/jcm9051419

**Published:** 2020-05-11

**Authors:** Markus Gerber, Christian Imboden, Johannes Beck, Serge Brand, Flora Colledge, Anne Eckert, Edith Holsboer-Trachsler, Uwe Pühse, Martin Hatzinger

**Affiliations:** 1 Sport Science Section, Department of Sport, Exercise and Health, University of Basel, CH-4052 Basel, Switzerland; Serge.brand@unibas.ch (S.B.); flora.colledge@unibas.ch (F.C.); Uwe.puehse@unibas.ch (U.P.); 2 Psychiatric Services Solothurn, 4503 Solothurn, Switzerland; Christian.Imboden@pkwyss.ch (C.I.); Martin.Hatzinger@spital.so.ch (M.H.); 3 Private Clinic Wyss, 3053 Muenchenbuchsee, Switzerland; 4 Clinic Sonnenhalde, 4125 Riehen, Switzerland; Johannes.Beck@sonnenhalde.ch; 5 University Psychiatric Clinics (UPK), Center for Affective, Stress and Sleep Disorders, University of Basel, 4002 Basel, Switzerland; edith.holsboer@gmail.com; 6 Substance Abuse Prevention Research Center and Sleep Disorders Research Center, Kermanshah University of Medical Sciences, Kermanshah 6715847141, Iran; 7 University Psychiatric Clinics (UPK), Neurobiology Laboratory for Brain Aging and Mental Health, University of Basel, 4002 Basel, Switzerland; anne.eckert@upkbs.ch

**Keywords:** cortisol, depression, exercise training, physical activity, stress reactivity

## Abstract

Physical activity is associated with a favourable (blunted) cortisol stress reactivity in healthy people. However, evidence from experimental study and with psychiatric patients is missing. This study examines whether exercise training impacts on cortisol stress reactivity in inpatients with major depressive disorder (MDD). These new insights are important because the stress reactivity of healthy people and patients with severe symptoms of depression might differ. Methods: The study was designed as a randomized controlled trial (trial registration number: NCT02679053). In total, 25 patients (13 women, 12 men, mean age: 38.1 ± 12.0 years) completed a laboratory stressor task before and after a six-week intervention period. Nine samples of salivary free cortisol were taken before and after the Trier social stress test (TSST). Fourteen participants took part in six weeks of aerobic exercise training, while 11 patients were allocated to the control condition. While the primary outcome of the study was depressive symptom severity, the focus of this paper is on one of the secondary outcomes (cortisol reactivity during the TSST). The impact of aerobic exercise training was examined with a repeated-measures analysis of variance. We also examined the association between change in depression and cortisol response via correlational analysis. Cortisol reactivity did not change from baseline to post-intervention, either in the intervention or the control group. Participation in six weeks of aerobic exercise training was not associated with participants’ cortisol reactivity. Moreover, depressive symptom change was not associated with change in cortisol response. Aerobic exercise training was not associated with patients’ stress reactivity in this study. Because many patients initially showed a relatively flat/blunted cortisol response curve, efforts might be needed to find out which treatments are most efficient to promote a normalization of HPA axis reactivity.

## 1. Introduction

Major depression is among the most common and burdensome disorders worldwide. In Switzerland [1], as in other Western countries [2], more than 20% of the population are affected by major depression once in a lifetime. Currently, major depressive disorders (MDDs) constitute the leading cause of years lived with disability (YLDs) [3] and in 2030, MDDs are expected to cause the greatest number of disability-adjusted life years (DALYs) [4].

Depression is often referred to as a stress-related disorder [5]. In line with this notion, exposure to critical life events and chronic stress play an important role in the onset and course of MDDs [6]. Accordingly, most individuals who develop a depressive episode experience a major adverse life event before depression onset [7]. Chronic stress exposure also increases the risk of developing a new depressive disorder [8]. Moreover, scholars have highlighted that people with depressive disorders are more likely to perceive their lives as stressful [9].

The relationship between stress and depressive disorders has often been attributed to a dysregulation of the hypothalamic-pituitary-adrenal (HPA) axis [10,11], and it is expected that a normalization of HPA axis activity will lead to an improvement in depressive symptom severity [12,13]. In line with this notion, previous studies have shown that chronically high stress levels are associated with a dysregulated diurnal HPA axis activity [14], an increased cortisol awakening response [15], and an increased reactivity in response to experimentally induced stress [16,17]. Similarly, evidence suggests that MDD might be associated with a dysregulation of HPA axis activity [18]. For instance, researchers observed a hyperactivity of the HPA axis and a lower suppression of cortisol after a dexamethasone suppression test (DST) among individuals with MDD [19,20,21]. Nevertheless, findings have not always been consistent [20]. For instance, in a systematic review and meta-analysis with 20 case-control studies, Knorr et al. [22] were unable to detect a clear difference in diurnal salivary cortisol levels between depressed patients and non-depressed controls, and some studies found that patients might even have lower cortisol levels [23,24]. The latter has been interpreted as an indication of HPA axis exhaustion, following chronic or recurrent depression [23,24].

Researchers have also tested whether people with depression differ from non-depressed counterparts with regard to their stress reactivity. If exposed to an acute stressor, healthy people generally show a dynamic and responsive pattern of cortisol secretion, with stress reactivity typically peaking 20 to 40 min after onset of the stressor [25]. In previous research, different patterns of cortisol secretion were found between depressed and non-depressed participants when they were exposed to an experimentally induced psychosocial stressor task [26]. However, the pattern seems to be moderated by a variety of factors, including participants’ age. Thus, in child and adolescent samples, no clear pattern emerged. While depressed participants exhibited a higher cortisol reactivity and a delay in cortisol recovery in some studies [27,28], the findings of other investigations pointed towards a blunted cortisol reaction [29,30]. Among adults, a meta-analysis indicated a similar cortisol reactivity, but higher pre-stress cortisol levels and increased cortisol levels during the recovery period in clinically depressed individuals compared to non-depressed individuals [31]. A more recent meta-analysis [32] with 12 studies focusing on HPA axis responses to the Trier social stress test (TSST: 33) (or comparable distressing paradigms) confirmed that depressed participants showed a reactivity pattern similar to that of healthy controls, after controlling for cortisol levels prior to the TSST. However, the meta-analysis of Burke et al. [31] suggested that stress reactivity might depend on depression severity since participants with more severe depressive symptoms tended to show a reduced cortisol response (flat and unresponsive pattern of cortisol secretion) in response to laboratory stress. The notion of a blunted cortisol reactivity is in line with other experimental and naturalistic studies [32,33,34,35,36]. In summary, previous research suggests that the cortisol reactivity of people with depressive disorders may differ from healthy individuals, however these differences seem to depend on several extraneous factors. Lopez-Duran et al. [37] argued that a larger variability in stress reactivity might exist in depressed participants than healthy controls, which is in line with Peeters et al. [38], who observed a higher variability in circadian cortisol secretion among patients with MDDs compared to healthy controls, if participants were assessed in their everyday environment.

Meanwhile, scholars have identified several sources of individual variability in stress reactivity [39]. One such factor is participants’ physical activity and fitness level. As shown in a recent systematic review [40], about 60% of all existing studies suggest that higher physical activity and fitness levels are associated with an attenuated cortisol response to the TSST, one of the most widely used and validated laboratory stressors [25]. However, Mücke et al. [40] also showed that most evidence is based on cross-sectional observations, whereas longitudinal and experimental studies are lacking. The impact of physical activity and fitness on adrenocortical activity has been explained with the so-called cross-stressor-adaptation (CSA) hypothesis [41]. This hypothesis suggests that exposure to physical stress (e.g., vigorous exercise) elicits a comparable stress reaction as the one following exposure to psychosocial stressors. With other words, the CSA posits that the (favourable) adaptations of HPA axis activity due to regular exercise participation generalize to situations where people are confronted with other, non-physical (e.g., cognitive or psychosocial) stressors. So far, however, only one experimental study has been carried out to test the CSA, with the TSST as an experimental stress trigger [42]. In this study, physically inactive (but generally healthy) male office workers recruited from banking and insurance companies were assigned to a 12-week running training program or a wait-list control condition. The findings of this study show that the cortisol stress reactivity decreased from pre- to post-intervention in the running group, which was not the case in the wait-list control group. While this study indicates that regular exercise training may indeed lead to a reduced stress reactivity, it is premature to draw firm conclusions about cause and effect until more experimental evidence has been collected. In view of the potential differences between healthy people and individuals suffering from MDDs, it also remains unclear whether these findings can be generalized to other (less healthy) populations.

A better understanding of whether such a generalization is possible seems important for several reasons: First, recent meta-analyses have shown that exercise training has the potential to decrease symptom severity [43,44,45,46,47,48,49] and to improve physical fitness [50] among patients suffering from mild to more severe depression. Second, findings from single trials suggest that exercise training may have effects comparable to those of pharmacological treatment [51], that exercise training has a positive impact if used as an add-on to standard care (compared with standard care alone) [52], and that even treatment-resistant patients might benefit from regular exercise training [53]. Third, researchers have shown increasing interest in the neurobiological mechanisms that may explain the benefits of exercise training in individuals with MDD [54,55]. For instance, Schuch et al. [54] recently argued that the benefits associated with exercise might be due to both acute and chronic effects, and be attributable to a variety of mediating factors including atrial natriuretic peptide, brain natriuretic peptide, copeptin, cortisol, growth hormone, prolactin, brain-derived neurotrophic factor, insulin like growth factor 1, vascular endothelial growth factor, pro-inflammatory cytokines, anti-inflammatory cytokines, oxidative stress, and changes on cortical structure and activity. In their review, Kandola et al. [55] also identified neuroendocrine response, neuroplasticity, inflammation and oxidative stress as the main underlying biological mechanisms. With regard to the neuroendocrine response, Kandola et al. [55] argued that prolonged and excessive cortisol exposure can have neurotoxic effects, lead to unfavourable changes of brain structures, and to cognitive deficits that are linked with depression. Accordingly, they conclude that “interventions that normalise HPA axis tone may minimise the corresponding neural harms and support the treatment of depression” (p. 529). This is in line with Matta Mello Portugal et al. [56] and Wegener et al. [57], who suggest that exercise training might result in precisely such a normalisation of the HPA axis activity.

Despite this, the evidence regarding the question of whether exercise leads to neuroendocrine adaptations in patients with MDD is still very limited. Kiive et al. [58] observed no differences with regard to the initial cortisol levels and responses to an incremental bicycle ergometer test between clinically depressed patients and healthy controls. This was partly corroborated by Krogh et al. [59], who found that resting plasma levels of cortisol did not differ between clinically depressed patients and healthy controls. However, their findings indicated that depressed patients showed a blunted cortisol response during an incremental bicycle ergometer test. Ida et al. [60] found that saliva free cortisol levels decreased after a single 15-min aerobic exercise session among depressed patients; and this decrease was accompanied by an improvement of subjective depressive symptoms. Krogh et al. [59] further reported that changes in cortisol levels did not differ between patients assigned to either 12-week exercise training or an active control condition (relaxation exercises). Nevertheless, this contrasts with a study by Foley et al. [61], who found that 12 weeks of aerobic exercise training resulted in a decreased cortisol awakening response (CAR), whereas the active control condition (stretching) was associated with an increased CAR. This is in line with the findings of Krogh et al. [59], who observed a significant decrease in the stress hormone copeptin [62] in the aerobic exercise condition (compared to the active control), particularly among patients with high compliance to the exercise intervention [63].

Taken together, an inadequate cortisol secretion as a consequence of a disturbed HPA axis activity seems to play an important role in the onset and maintenance of MDD [64]. Cortisol is considered as a mediating factor linking chronic psychosocial stress with depression [5]. Although chronic stress and exercise both trigger a cortisol response [65,66], chronic stress has a detrimental effect on cognition, neural plasticity and well-being, whereas exercise seems to have a beneficial impact [67]. It is therefore possible that both acute and chronic exercise might contribute to a normalization of HPA axis activity [56,57]. However, research in patients with MDD is very limited, and results are not conclusive. Recent reviews therefore collectively come to the conclusion that more research is needed in this area [54,68,69]. Although the TSST is the most widely used laboratory stressor to experimentally induce a stress response [70], and previous studies suggest that regular exercise participation might positively affect the cortisol response to the TSST among healthy people [40,42], the effects of chronic exercise on the cortisol reactivity in response to the TSST have not yet been tested among patients with MDD.

Against this background, the main purpose of our study was to test for the first time whether an exercise intervention has an impact on cortisol stress reactivity in a psychiatric patient population (inpatients with MDD). Based on the existing body of research, it was not possible to formulate a clear-cut hypothesis: While the only existing exercise trial with healthy participants indicates that exercise training results in a blunted cortisol secretion in response to the TSST [42], evidence with participants with more severe depressive symptoms suggests that this population shows a flat and unresponsive pattern of cortisol secretion in response to laboratory stress. If the cortisol response is blunted, it seems unlikely that a further reduction in cortisol response as consequence of exercise training will be observable. In this case, it might also be that exercise training may lead to a normalization of cortisol secretion under laboratory stress conditions.

## 2. Materials and Methods

### 2.1. Participants and Procedures

Participants were recruited from two (public) psychiatric clinics in the Northwestern, German-speaking part of Switzerland. The following inclusion criteria were assessed by the consultant psychiatrist of the respective depression ward as part of the initial screening (in the first week of hospitalization), prior to providing more specific information about the study to the eligible patients: (a) aged >18 and <61 years, (b) be an inpatient in one of the two depression wards, (c) ICD-10 diagnosis of depression (first episode, recurrent or bipolar; F32, F33, F31). Moreover, the following exclusion criteria were applied: (a) a score <17 on the Hamilton depression rating scale 17 (HDRS17) at the baseline data assessment, (b) presence of a somatic condition not permitting regular exercise, (c) body mass index (BMI) > 35 kg/m^2^, (d) pregnant at the baseline data assessment, (e) acute suicidal ideation, (f) comorbid substance dependence (except nicotine), (g) comorbid major psychiatric disorder, and (h) participation in regular vigorous-intensity exercise (i.e., participating in long distance runs).

The study was designed as a two-centre, two-armed randomised controlled trial (RCT), with a 1:1 allocation ratio (intervention:control group). The study was registered at https://www.clinicaltrials.gov (trial number: NCT02679053). As highlighted in the study protocol [71], the primary outcome of this study was depression severity. While the effects of the intervention on the primary outcome are presented elsewhere [72], the focus of the present paper is on one of the secondary outcomes (cortisol response during the TSST).

**Figure 1:**
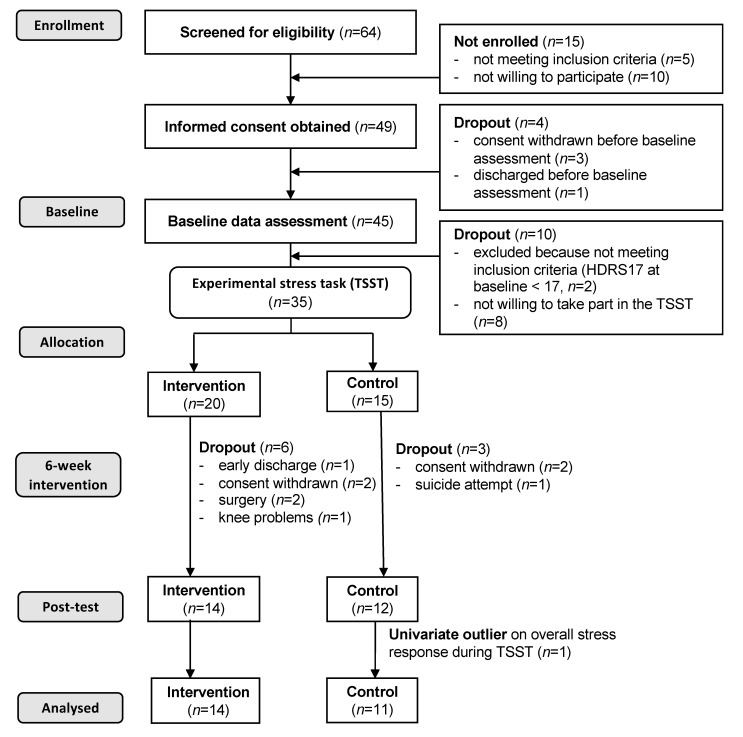
Participant flow chart.

Recruitment took place between October 2013 and January 2016, and was done by the local study coordinators. A graphical representation of the study schedule including information about which measurements were performed at which timepoint is presented in the study protocol [71]. As shown in Figure 1, written informed consent was obtained from all participants that were eligible for the study, and they were allocated to either an aerobic exercise condition (intervention group) or a standardised stretching and mobility program (active control group) after the baseline data assessment. The last author was responsible for the randomisation and assignment of the participants to the intervention and control group. Simple randomisation was achieved by drawing lots for the intervention and control condition, separately for males and females. Blinding of patients with regard to group allocation was achieved by informing them that two different exercise programs were to be compared, but not letting them know which intervention was considered the intervention or control condition. Data assessors and local study coordinators were fully blinded to group allocation (by anonymized ID). The baseline data assessment took place during the first or second week of hospitalization.

To detect a moderate effect (f = 0.25) in the RCT on the primary outcome (depressive symptoms severity), a power analysis for repeated measures analyses of variance (ANOVAs) (using G*Power 3.1; alpha error probability: 0.05, power: 0.90, correlations among measures: 0.50) revealed that at least *n* = 36 (overall) participants are needed. We therefore originally aimed to include 40 patients with a roughly equal gender distribution.

Before the beginning of the study, ethical clearance was obtained from the Ethics Committee of both Basels (EKBB, Basel, Switzerland; reference no. 62/13) and the Ethics Committee Aargau/Solothurn (Aarau, Switzerland; reference no. 2013/029) and all study procedures were carried out in line with the ethical principles defined in the 1964 Declaration of Helsinki and its later amendments. 

### 2.2. Intervention vs. Control Condition

The intervention consisted of supervised aerobic exercise on indoor bicycles three times per week for six consecutive weeks. The target heartrate (HR) was set at 60–75% of maximal heartrate (HRmax) monitored with Polar™ RS800CX. We used the following formula (220–age (in years)) to compute HRmax. Following Dunn et al. [73], the targeted exercise-based energy expenditure was 17.5 kcal per kg bodyweight. 

Participants assigned to the active control condition, engaged in a program consisting of coordination and stretching activities for all major muscle groups using a medium strength Theraband^®^, a gymnastics ball (diameter 65 cm) and juggling balls, which also took place three times per week for six consecutive weeks. To ensure that the intensity in the control group was kept at a low level during the stretching sessions, supervisors reminded the participants of the control group that they should not get out of breath. This was important to avoid an overlap in activity intensity between the intervention and the control group. To minimize the influence of social contact, the coordination and stretching activities were also carried out individually or in groups of two patients.

All sessions (intervention and active control group) were scheduled in the late afternoon (between 4 and 6 p.m.) for approximately 40–50 min. Additionally, all patients received standard inpatient treatment consisting of pharmacological treatment according to Swiss national standards [74], individual and group-psychotherapy supported by an array of creative group therapies. Pharmacological treatment was limited to antidepressant treatment with selective serotonine-reuptake-inhibitors (SSRI) or selective serotonine-norepinephrine-reuptake-inhibitors (SNRI) and lithium as augmentation strategy, whereas antidepressant combination therapy, tricyclic antidepressants, MAO-inhibitors, and antipsychotics other than low-dose quetiapine for sedation were not allowed. Participants were asked not to engage in any additional vigorous exercise activities during their stay at the hospital. However, engagement in additional vigorous exercise was not systematically monitored. Evidence regarding compliance with the intervention program has been reported previously [71]. 

### 2.3. Trier Social Stress Test

We used the Trier Social Stress Test (TSST) [33] to experimentally induce stress. The TSST consists of two standardised 5-min tasks including a free speech task (job interview) and a mental arithmetic task (counting backwards in steps of 13 from a 4-digit number). At both measurement occasions, the TSST was performed in front of a jury of two people unknown to the patient. Patients were also informed that the test would be videotaped to evaluate their behaviour. Patients had 5 min to prepare themselves for the speech task, before they entered the test room. The test was completed in a standing position. After completion of the TSST, participants recovered in a separate room for a period of 60 min. In the present study, the TSST was carried out exactly at 2 p.m. to control for circadian variations of cortisol levels [75]. The participants were also asked to refrain from eating, drinking (except for water) and from any moderate or vigorous intensity physical activity 2 h prior to the beginning of the stress protocol. While previous research has shown that participation in physical activity prior to the TSST might have acute effects on stress reactivity [76], researchers also showed that cortisol levels typically return to the level prior to the TSST within two hours after individual have stopped exercise participation [77]. All participants were unaware of the experimental protocol. Because stress reactions depend on the novelty of the stressor task [25], responses might be less strong if the same psychosocial stressor task is performed a second time [78]. However, researchers showed that the stressfulness of the TSST can be maintained if the stressor tasks are modified [79]. We therefore slightly changed the content of the free speech task (application for a promotion/prize) and mental arithmetic tasks (counting backwards in steps of 13 from a different number), but ensured that the socio-evaluative pressure of the stressor protocol was maintained. According to Mazurka et al. [80], the TSST is useful for examining the stress reactivity in depression “because it provokes the HPA axis response by activating the same corticolimbic circuitry implicated in the pathology of major depression” (…), whereas “pharmacologic challenge tests and studies of the cortisol awakening response bypass this important circuitry and directly activate components of the axis” (pp. 301–302).

### 2.4. Assessment of the Adrenocortical Stress Response

The adrenocortical stress response was measured via salivary cortisol samples. To minimize an anticipation bias, we gathered the first saliva sample before participants were informed about the nature of the experimental stress protocol. At both the baseline data assessment and at post-intervention, salivary cortisol was obtained at −20, −5, +1, +5, +10, +20, +30, +45, and +60 min prior to the onset/after completion of the TSST, using commercially available devices (Salivette®, Sarstedt, Germany). All samples were stored at −20 °C, before they were sent to the Biochemical Laboratory of the University of Trier for analyses (approximately every three months). Free salivary cortisol levels (nmol/L) were established, based on a time-resolved fluorescence immunoassay (for more details see: [81]).

### 2.5. Assessment of Covariates

#### 2.5.1. Depressive Symptom Severity

Symptom severity of depression was measured with the 21-item Beck Depression Inventory [82,83]. The BDI measures a range of affective, behavioural, cognitive, and somatic symptoms, which are characteristic for unipolar depression (e.g., “I am so unhappy/sad that I can’t stand it.”). Response options are anchored on a scale from 0 to 3, with higher scores being indicative of stronger depressive symptomatology. A sum score was calculated to generate an overall index with a potential range from 0 to 63 and higher scores reflecting more severe depressive symptoms. Scores between 0 and 9 are considered as no depression, whereas scores of 10–18, 19–29, and 30–63 can be interpreted as mild, moderate and severe depression, respectively. The Cronbach’s alpha in the present sample was 0.72 at baseline. 

#### 2.5.2. Physical Activity

To gather information about participants’ physical activity levels prior to hospitalization, participants filled in a written version of the German International Physical Activity Questionnaire Short Form (IPAQ-SF) [84]. The IPAQ-SF assesses time spent in moderate physical activity (e.g., bicycling at a regular pace, low-intensity sports such as doubles tennis) and vigorous physical activity (e.g., fast bicycling, running, aerobics), using a frequency-by-duration format. Thus, participants first reported the number of days per week they engaged in these activities (from 0 to 7 days), and then indicated the average duration (in minutes) for the days they engaged in these activities. Participants were asked to refer to the last week prior to admission to the hospital. Multiplication of frequency and duration scores resulted in an estimate of weekly hours invested in moderate and vigorous physical activity. Summing up these two scores resulted in a total score for time (in min/week) spent in moderate-to-vigorous physical activity (MVPA). Evidence for the validity of the IPAQ-SF has been shown previously in adult samples [85].

#### 2.5.3. Further Potential Confounders

We assessed sex, age, smoking status, educational status, duration of current depressive episode, number of prior depressive episodes, and age of onset of depression. Additionally, all patients underwent a physical examination including the assessment of blood pressure (BP), resting heart rate (HR), as well as body weight and height. Body weight (kg) and height (m) were used to calculate participants’ body mass index (BMI; kg/m^2^).

### 2.6. Statistical Analyses

We first examined whether results of the different variables are normally distributed (using Kolmogorov–Smirnov and Shapiro–Wilk tests). To examine whether baseline differences existed between the intervention and control groups, in the case of normally distributed (metric) variables, ANOVAs were conducted, and descriptive statistics were reported as M and SD. If either the Kolmogorov–Smirnov or the Shapiro–Wilk test indicated a non-normal distribution of a metric variable, nonparametric tests were used (independent samples median test (Kruskal–Wallis) and independent samples Mann–Whitney U Test). Moreover, in case of non-normally distributed (metric) variables, we reported the median (Mdn) and range (min; max). For categorical variables, we used χ^2^ tests to examine group differences, and we reported the number (*n*) and frequency (%) as descriptive statistics. 

To assess bivariate associations between the potential covariates and the cortisol responses at baseline and post-intervention, correlational analyses were conducted. Pearson’s correlations (r) were used if covariates were normally distributed. Spearman’s correlations (Rho) were used if covariates were non-normally distributed. In case of non-normally distributed cortisol values, cortisol scores were logarithmized to achieve a normal distribution prior to calculating the correlations. Moreover, in case of categorical covariates, analyses of variance (ANOVAs) was conducted.

To examine whether the TSST elicited a significant stress response in the total sample and the intervention and control groups, a repeated measure analysis of variance (ANOVA) was calculated, with the factors time (two measures from 20 min prior to the onset of the TSST to 20 min after completion of the stressor task (peak) and group (intervention vs. control group), and the interaction effect (time x group). The time point 20 min after completion of the stressor task was chosen as a peak, as most studies show that cortisol levels tend to increase approximately 20 min after the end of a stressor task [40]. Again, in case of non-normally distributed cortisol values, the logarithmized scores were used. We also descriptively analysed how many participants responded to the TSST, hereby following Petrowski et al. [86] who defined a response to the TSST as a salivary cortisol increase of ≥ 2.5 mmol/L from prior to the TSST to the individual peak.

As an index of overall cortisol stress reactivity, we calculated the area under the total response curve with respect to ground (AUC_G_), by using the trapezoid formula described by Pruessner et al. [87], including the entire time period from 20 min prior to the onset to 60 min after completion of the TSST. We did not use the total response curve with respect to increase (AUC_I_), because this index results in negative values if the repeated measures are lower than the cortisol level prior to the TSST.

To examine whether six weeks of aerobic exercise training had an effect on participants’ overall stress reactivity, we employed a repeated measures analysis of covariance (ANCOVA) with AUC_G_ as an outcome, with the factors time (baseline vs. post-intervention), group (intervention vs. control group), and the interaction effect (time × group), and controlling for depressive symptom severity, physical activity and further relevant covariates (age, depressive symptom severity, duration of current depressive episode, number of prior depressive episodes, age at onset of depression, resting heart rate, blood pressure, height, weight, BMI, MVPA, sex, smoking status, educational background, and diagnoses). However, covariates were only considered if they were significantly associated with the cortisol stress response either at baseline or post-intervention. To interpret the magnitude of the effects, η^2^ values were shown for both main and interaction effects. Finally, we calculated a Pearson’s correlation to examine whether changes in depressive symptom severity were associated with changes in cortisol response. The level of significance was set at *p* < 0.05 across all analyses. All tests were carried with SPSS 24 for Mac (SPSS Inc., Chicago, IL).

## 3. Results

### 3.1. Sample Characteristics and Descriptive Statistics

As shown in the participant flow chart (Figure 1), 49 participants (25 women, 24 men, M = 38.9 years, SD = 11.3) were willing to take part in the study. Thereof, four participants (three women, one man, M = 33.5 years, SD = 13.2) dropped out before the beginning of the data assessment (three withdrew consent, one was discharged). Of the remaining 45 participants, 10 participants (five women, five men, M = 37.0 years, SD = 9.8) were not willing to take part in the experimental stress test (*n* = 8) or did not meet the inclusion criteria with regard to depression severity (*n* = 2). Of the remaining 35 participants with valid baseline data, nine dropped out before post-intervention due to the following reasons: early discharge (*n* = 1), withdrawn consent (*n* = 4), surgery (*n* = 2), knee problems (*n* = 1), and suicide attempt (*n* = 1). The attempted suicide was considered as a severe adverse event and immediately reported to the local ethical review committee. However, since there was no reason to believe that the suicide attempt was caused by the study intervention, no further actions were deemed necessary other than a more rigorous assessment of suicidal behaviour prior to study inclusion. One participant was identified as a univariate outlier because stress reactivity exceeded three standard deviations around the mean. This person was excluded from all further analyses. Among the patients who took part in the first TSST assessment, no significant differences were found between dropouts and study completers in any of the study variables. The post-intervention assessment took place immediately after completion of the six-week intervention/control period.

As shown in Table 1, not all (metric) study variables were normally distributed in the present sample. This is taken into consideration when sample characteristics and descriptive statistics for the baseline and post-intervention data assessment are presented (Table 1).

On average, the total sample (*n* = 25) was 38.1 (SD = 12.0) years old, and consisted of 13 women and 12 men. Most of the participants were diagnosed with a first unipolar depressive episode (*n* = 10, 40%) or recurrent depressive episode (*n* = 14, 56%); only one participant (4%) was diagnosed with bipolar depression. Based on the BDI scores, three participants (12%) reported mild depression, 14 (56%) reported moderate depression, and eight (32%) reported severe depression. Based on participant self-reports, five participants (20%) reported that they were completely inactive (0 min/week of MVPA) prior to hospitalization. Fourteen participants (56%) reported some MVPA, but according to the standards of the American College of Sports Medicine [88], did not meet current MVPA recommendations (≥150 min/week). Six participants (24%) accomplished these standards. As shown in Table 1, no significant baseline differences were found between participants assigned to the intervention and control group in any of the study variables.

### 3.2. Reactivity in Response to the TSST at the Baseline Data Assessment

**Figure 2:**
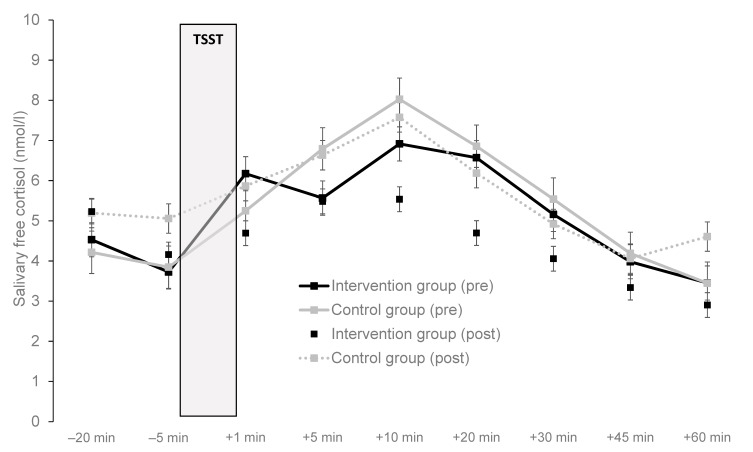
Stress reactivity pattern before and after six weeks of exercise training, in the total sample, the intervention group and the control group, from 20 min prior to the onset of the TSST until 60 min after completion of the TSST. Note. Error bars represent the standard errors (SE).

At the baseline data assessment, a repeated measures ANOVA showed that in the total sample, a significant time effect occurred prior to the onset of the TSST (−20 min) to peak (+20 min after completion of the TSST), F(2,23) = 4.4, *p* = 0.046.05, η^2^ = 0.161, indicating that the TSST elicited a cortisol response. However, no significant time x group interaction effect was observed, F(2,23) = 0.1, *p* = 0.769, η^2^ = 0.004, and the response curve was flat in both the intervention and control group (see Figure 2). At the baseline assessment, only a 1.6-fold increase was observed from 20 min prior to the TSST to peak in the total sample (see Table 1). Moreover, at the baseline data assessment, 52 percent (*n* = 13) of the participants responded to the TSST (increase in cortisol from 20 min prior to the TSST to individual peak of ≥2.5 mmol/l), whereas 48 percent of the participants were classified as non-responders (*n* = 12).

### 3.3. Associations Between Potential Confounders and Stress Reactivity

As shown in Table 2, bivariate correlations and univariate ANOVAs revealed that none of the potential confounders was significantly associated with the overall cortisol stress response during the TSST (neither at baseline nor at post-intervention), as measured with AUC_G_. Therefore, none of these factors were considered as a potential confounder in the subsequent repeated measures ANOVAs.

### 3.4. Impact on Aerobic Exercise Training on Stress Reactivity

The results of the repeated measures ANOVAs, addressing the main study question, are displayed in Table 3. No significant main and interaction effects occurred. As shown in Figure 2, no major differences were found in cortisol response pattern at baseline and post-intervention between patients of the intervention and control group.

### 3.5. Correlations of Change Between Depressive Symptoms and Cortisol Response

Although not the focus of the present paper, Table 3 shows that depressive symptom severity considerably decreased from baseline to post-intervention in both the intervention and the control group. Accordingly, a strong main effect for time was found in the repeated measures ANOVA. However, no significant time × group interaction was found in the present sample.

To examine the relationship between change in depressive symptom severity and change in cortisol response, a Pearson’s correlation was computed (based on Kolmogorov–Smirnov and Shapiro–Wilk tests, both change scores were normally distributed). The Pearson’s correlation between these variables was r = 0.02, *p* = 0.944.

## 4. Discussion

The present study shows that six weeks of exercise training did not result in an altered pattern of cortisol secretion in response to an acute psychosocial stressor among inpatients with MDD. The findings are novel because this is the first study worldwide testing the validity of the cross-stressor adaption (CSA) hypothesis in psychiatric patients [40]. Moreover, only one further intervention trial exists in which the impact of an exercise training program was tested on the cortisol reactivity to the TSST [42]. Changes in depressive symptom severity were not correlated with changes in cortisol response to the TSST.

In contrast with the work of Klaperski et al. [42] with generally healthy, but physically inactive employees, regular aerobic exercise training did not result in a blunted stress reactivity in our sample. Figure 2 indicates that, in our sample, the cortisol response pattern remained nearly unchanged from pre- to post-intervention in both participants assigned to the intervention and control condition. This is remarkable as depressive symptom severity considerably decreased in both the intervention and control group between the two assessment periods. A more detailed discussion regarding the effects of the intervention on the primary outcome (depression symptom severity) is presented elsewhere [72]. Obviously, changes in depressive symptom severity and cortisol secretion during the TSST do not change in parallel among patients with MDD undergoing inpatient treatment. While our findings suggest that exercise training is more suited to impact on stress reactivity among healthy people, these results do not allow too far-reaching conclusions. As highlighted in the introduction section, research on the neurobiological mechanisms that explain the benefits of exercise training in individuals with MDD is still in a very early stage [69], and research with neuroendocrine markers is sparse [54,68]. Therefore, further research is needed to find out whether different effects occur if the training is maintained over longer periods of time, and if patients with differing psychiatric disorders are assessed.

Beyond the main research question addressed in this paper, some further observations were made. Our first observation was that as reported in the meta-analysis by Burke et al. [31], patients with MDD tend to show a relatively non-responsive cortisol secretion pattern if exposed to the TSST. This notion was supported in the present study. Although patients exhibited a significant increase in free salivary cortisol concentration, on average, we found only a 1.6-fold increase from 20 min prior to the TSST to peak, which is considerably lower than the response to socio-evaluative stressors in healthy populations [25]. Moreover, the median cortisol concentrations at the peak (+20 min after completion of the TSST) varied between 5.2 nmol/L (at the baseline data assessment) and 4.7 nmol/L (at post-intervention) in the total sample. Again, this concentration is lower compared to studies with healthy participants where group peak cortisol concentrations reached levels of >15 nmol/L [33,89]. Moreover, no evidence was found that patients’ non-responsiveness was due to heightened pre-stress cortisol concentrations. As a putative mechanism for the blunted cortisol reactivity, Suzuki et al. [90] pointed towards a downregulation of HPA axis activity among patients with MDD [91]. As highlighted by McEwen and Stellar [92] and Juster et al. [93], exposure to chronic stress might lead to a repeated overactivation and impairment of the HPA axis. This notion is in line with the fact that chronic stress and critical life events play a major role in the development and maintenance of MDD [5,6]. This finding raises important questions regarding the interpretation of low cortisol responses. Thus, while a low stress reactivity to acute laboratory stressors is generally regarded adaptive among healthy participants [94,95], this is not necessarily the case among patients with MDD. In a model focussing on brain structures involved in the regulation of stress reactivity, Lovallo [96] argued that a dysregulated stress reactivity on the central nervous level (prefrontal cortex, limbic system, hypothalamus and brain stem) will lead to poor behavioral homeostasis. Accordingly, Lovallo assumes that stress reactivity may range from very low to very high and that both exaggerated and diminished stress reactivity may have a negative impact on health, because both tendencies are indicative of a loss of homeostatic regulation.

Our second observation was that self-reported physical activity levels prior to hospitalization were not associated with patients’ cortisol responses (either at the baseline data assessment or at post-intervention). This is in contrast to previous results with healthy population, in which the CSA hypothesis associated with physical activity and fitness was supported in about 60% of all studies [40]. The lack of a significant relationship can be attributed to various factors. First, it is difficult to detect significant differences in a non-responsive sample. Second, most of the participants in our sample were physically inactive or reported low physical activity levels. Thus, individual variability was limited in the independent variable, as well.

Our third observation was that physical activity levels were relatively low among patients with depressive disorders. In a previous meta-analysis, Schuch et al. [97] highlighted that among patients with MDD, more than two thirds (68%) did not reach current levels of recommended physical activity (≥150 min of MVPA per week). Moreover, Schuch et al. [97] showed that patients with MDD were significantly more likely to miss MVPA recommendations than healthy controls. In the present study, only 18% of the participants reached recommended MVPA standards. Given this background, it is encouraging that sport and exercise training are increasingly used in Swiss psychiatric clinics as an additional treatment approach [98], and that many MDD patients are sufficiently physically active during their stay at the clinics [99]. Nevertheless, more systematic efforts are needed to increase patients’ lifestyle physical activity sustainably [100,101], because the beneficial effects of exercise and sport therapy may dissipate if the training is stopped after discharge from the clinic [102,103].

Finally, in contrast to previous studies [104], we did not find a significant association between BMI and participants’ cortisol reactivity. Moreover, we did not find a link between age and stress reactivity although previous studies suggested that the level of HPA axis dysregulation might increase with age among people with depression [20,21]. We also did not observe a difference in cortisol stress reactivity between women and men. While it is well established that women are more prone to develop depressive symptoms and MDD compared to men [105], studies examining sex differences in stress reactivity have yielded equivocal findings [106], with men responding more strongly [107], less strongly [108] or similarly [109] if compared to female participants. Chopra et al. [110] found in a study comparing patients with chronic MDD with healthy controls that female patients exhibited a higher reactivity, whereas a blunted reactivity was found in male patients. Nevertheless, the opposite pattern of results was found in a study with depressed and non-depressed adolescents [80]. Thus, depressed boys showed a higher cortisol reactivity than depressed girls, and the latter had a blunted cortisol response in comparison to non-depressed girls.

The strengths of the present study can be summarized as follows. As mentioned previously, this is the first study testing the CSA hypothesis in psychiatric patients, a population in which HPA axis activity is often dysregulated. In the present study, all participants were diagnosed with MDD according to ICD−10 definitions, were in-patients, and most of them reported moderate-to-severe levels of depression. This is noteworthy, as Burke et al. [31] showed that cortisol reactivity is particularly low in in-patients and in more severely depressed people. Moreover, few intervention studies have been carried out so far to examine whether exercise training positively impacts on stress reactivity. Another strength was that we first tested bivariate relationships with potential confounders to rule out that these factors confound the association between self-reported physical activity and cortisol reactivity. We used an established psychosocial stressor, which is known to trigger a stronger stress response compared to other cognitive stressors due to its socio-evaluative character. Gender distribution was almost equal in our sample. Finally, we systematically assessed energy expenditure in the intervention group, showing that the compliance rate was high in the intervention group [71]. 

Despite these strengths, our findings need to be interpreted in light of several limitations. First, the sample size was small. Thus, the low sample size did not allow for a consideration of further moderating factors (such as sex, baseline physical activity levels, symptom severity, etc.). For instance, we did not control atypical depression, a specific type of depression that has been associated with stronger appetite, hyperphagia and hypersomnia, and a lower stimulation of the HPA axis [111]. We also acknowledge that our power calculation was based on the primary outcome (depressive symptom severity) and not on cortisol response, although these are two different outcomes. Moreover, a posteriori power analyses showed that with a repeated measures design (within-between interaction, alpha error = 0.05, Power = 0.80, two groups, two measurements, correlations among repeated measures of r = 0.35), we would have needed a total sample size of 258 participants to detect small effects (f = 0.1) or 44 participants to detect moderate effects (f = 0.25). Because we only included 25 patients, our study was somewhat underpowered. Second, we were not able to control all influences that may have an influence on individuals’ stress response (such as previous critical life events). Moreover, for future studies with a repeated-measures design using two slightly different forms of the TSST, it seems advisable to assign half of the group to the modified test at pre-intervention and then switch tests at post-intervention to rule out the possibility that different tests produce different stress reactions. Third, we also acknowledge that physical activity was based on participants’ self-reports that might be negatively biased, especially since we studied a clinically depressed population. Fourth, the intervention period was relatively short (six weeks). Thus, exercise training might have had a stronger effect if carried out across a longer period of time. Fifth, a considerable number of eligible participants were not willing to take part in the TSST, or dropped out from the baseline data assessment to post-intervention, which may have entailed a selection and dropout bias. Nevertheless, we did not find substantial differences between patients who declined participation, dropouts and patients who volunteered for the TSST. Moreover, while we initially employed multiple imputation to substitute missing values, this yielded highly improbable values, which we attributed to the small sample size. We therefore proceed with a “per-protocol analysis” and did not carry out intention-to-treat analyses, as originally foreseen in the study protocol [71]. Sixth, our study does not allow a direct comparison with healthy controls. Such analyses may be important to more definitely establish whether the validity of the CSA hypothesis holds in both psychiatric and healthy participants. Seventh, our sample included a fairly broad age-range. Eighth, we did not systematically assess the physical activity levels of participants in the control group during the intervention period. Thus, we cannot exclude that these patients also engaged in moderate-to-vigorous physical activities while they were hospitalized. Ninth, we acknowledge that our control group also got some form of physical activity intervention (standardized coordination and stretching activities). While the instructors made efforts to keep the intensity level low, we did not monitor the intensity level systematically in this group. Moreover, an additional “treatment as usual” (TAU) control group could have been helpful to further assess the effect of the aerobic exercise intervention and the active control condition. Tenth, although we have carried out a six-month follow-up assessment in order to assess the longer-term effects on the primary and secondary outcomes, due to limited resources, we had to refrain from running the TSST a third time. Finally, no generalization is possible to other stress reactivity indicators (e.g., cardiovascular, emotional) and to stress reactivity in real-life conditions. The latter is important as researchers have shown that cortisol reactivity following the TSST and in response to a real-life stressor are only weakly related. In line with this notion, scholars assumed that stress reactions might be less strong in a laboratory setting [112], because emotional involvement is limited during an experimental stressor test [113].

## 5. Conclusions

Patients with MDD tend to self-report insufficient physical activity levels and show a blunted cortisol response pattern in following socio-evaluative stress. Therefore, systematic efforts are needed to increase participants’ physical activity levels and foster their cardiorespiratory fitness and cardiovascular health. More research is needed to find out which treatments are most efficient to promote a normalization of HPA axis reactivity in patients with MDD. The present study suggests that six weeks of aerobic exercise training are insufficient to impact on patients’ cortisol stress reactivity. Nevertheless, since this was the first study on the CSA hypothesis with psychiatric patients, the findings of our randomized controlled trial need to interpreted with caution until a broader empirical evidence base exists.

## Figures and Tables

**Table 1:** Sample characteristics and descriptive statistics of participants (*n* = 25) with complete baseline and post-intervention data.

	Total Sample (*n* = 25)		Intervention Group (*n* = 14)		Control Group (*n* = 11)		Test of between-Group Differences (ANOVAs)				
Metric variables (normally distributed)	M	SD	M	SD	M	SD	F	*p*			η^2^
Age	38.1	12.0	39.4	9.7	36.4	14.8	0.39	0.539			0.017
Depressive symptom severity (BDI) at baseline	26.4	8.6	27.6	10.0	24.9	6.6	0.61	0.443			0.026
Depressive symptom severity (BDI) at post-intervention	15.7	11.1	17.1	12.5	14.0	9.2	0.46	0.503			0.020
Age at onset of depression	33.1	12.9	33.8	12.1	32.2	14.5	0.09	0.766			0.004
Resting heart rate (bpm)	75.0	12.8	78.1	14.3	71.0	9.8	1.95	0.176			0.078
Systolic blood pressure (mmHg)	123.9	18.2	128.4	18.9	118.3	16.3	1.97	0.173			0.079
Diastolic blood pressure (mmHg)	75.9	9.9	78.6	7.0	72.4	12.1	1.95	0.176			0.078
Height (cm)	172.1	8.1	171.6	7.4	172.7	9.3	0.12	0.732			0.005
Weight (kg)	71.6	20.3	74.1	22.2	68.4	18.2	0.47	0.500			0.020
BMI (kg/m^2^)	23.7	4.8	24.7	5.4	22.6	3.8	1.20	0.285			0.050
Metric variables (non-normally distributed)	Mdn	min; max	Mdn	min; max	Mdn	min; max	Independent Samples MedianTest *(p*-Value)		Independent Samples Kruskal-Wallis Test (*p*-Value)		
Duration of current depressive episode	12	3; 52	10	3; 52	13	5; 50	0.202		0.703		
Number of prior depressive episodes	1	0; 5	1	0; 5	1	0; 5	0.666		0.649		
Moderate-to-vigorous physical activity (min/week)	60	0; 540	60	0; 360	45	0; 540	0.609		0.582		
Cortisol level –20 min prior to TSST onset (nmol/L) at baseline	3.2	1.2; 8.8	3.0	2.1; 8.8	4.3	1.2; 7.9	0.609		0.582		
Cortisol level +20 min after completion of TSST (nmol/L) at baseline	5.2	1.1; 31.9	4.2	1.7; 31.9	8.1	1.1; 16.1	0.344		0.324		
Overall cortisol response (AUC_G_) at baseline	380.8	114.5; 1225.6	299.2	157.4; 1225.6	521.0	114.8; 805.0	0.267		0.250		
Cortisol level –20 min prior to TSST onset (nmol/L) at post-intervention	5.0	2.0; 13.9	4.7	2.0; 13.9	5.9	2.4; 9.1	0.687		0.661		
Cortisol level + 20 min after completion of TSST (nmol/L) at post-intervention	4.7	2.2; 21.4	3.0	2.2; 16.1	5.2	2.5; 21.4	0.095		0.090		
Overall cortisol response (AUC_G_) at post-intervention	392.3	200.2; 1252.0	351.1	200.2; 992.5	429.6	217.6; 1252.0	0.202		0.189		
Categorial variables	*n*	%	*n*	%	*n*	%	χ^2^			*p*	
Sex (females)	13	52	6	43	7	64	1.07			0.302	
Smoking status (smokers)	9	36	4	29	5	46	0.76			0.383	
Educational background											
Compulsory school	3	13	2	18	1	9	3.30			0.194	
High school	14	64	5	46	9	82					
Higher education	5	23	4	36	1	9					
Diagnoses											
F31 (Bipolar affective disorder)	1	4	1	7	0	0	0.90			0.625	
F32 (Unipolar depressive episode)	10	40	5	36	5	46					
F33 (Recurrent depressive disorder)	14	56	8	57	6	54					

Notes. Normal distribution was tested with Kolmogorov-Smirnov test and Shapiro-Wilk test. BDI = Beck Depression Inventory. bpm = Beats per minute. BMI = Body Mass Index. AUC_G_ = Area under the curve with respect to ground. TSST = Trier Social Stress Test. M = Mean. SD = Standard deviation. Mdn = Median.

**Table 2:** Bivariate associations between potential confounders and stress reactivity (AUC_G_) at the baseline data assessment and at post-intervention.

			Bivariate Correlations with AUC_G_^a^										
Metric Variables			Baseline					Post-Intervention					
			r/Rho			*p*		r/Rho			*p*		
Age ^b^			0.09			0.671		−0.07			0.744		
Depressive symptom severity (BDI) at baseline ^b^			0.17			0.430		−0.17			0.419		
Duration of current depressive episode ^c^			0.155			0.458		−0.10			0.631		
Number of prior depressive episodes ^c^			0.11			0.601		−0.35			0.095		
Age at onset of depression ^b^			−0.04			0.849		−0.05			0.817		
Resting heart rate (bpm) ^b^			−0.22			0.301		0.14			0.514		
Systolic blood pressure (mmHg) ^b^			0.09			0.662		0.04			0.856		
Diastolic blood pressure (mmHg) ^b^			0.28			0.184		0.26			0.214		
Height (cm) ^b^			0.34			0.091		0.20			0.337		
Weight (kg) ^b^			0.33			0.104		0.22			0.300		
BMI (kg/m^2^) ^b^			0.27			0.185		0.17			0.410		
Moderate-to-vigorous physical activity (min/week) ^c^													
	Group differences in AUC_G_^d^												
Categorial variables	Baseline						Post-intervention						
	M	SD	F	*p*	η^2^		M		SD	F		*p*	η^2^
Sex			2.0	0.174	0.100					0.0		0.937	0.000
Males	538	312					413		135				
Females	373	192					466		315				
Smoking status			1.3	0.268	0.053					0.0		0.836	0.002
Smokers	506	223					425		201				
Non-smokers	421	224					468		315				
Educational background			0.1	0.942	0.006					0.1		0.939	0.007
Compulsory school	421	274					405		172				
High school	467	197					480		300				
Higher education	573	440					413		153				
Diagnoses			0.6	0.537	0.055					0.1		0.909	0.009
F31 (Bipolar affective disorder)	720	0.0					440		0.0				
F32 (Unipolar depressive episode)	443	340					433		142				
F33 (Recurrent depressive disorder)	439	209					445		242				

Notes. BDI = Beck Depression Inventory. BMI = Body Mass Index. AUC_G_ = Area under the response curve with respect to ground of salivary free cortisol. Bpm = Beats per minute. ^a^ Correlations based on logarithmized AUC_G_ scores. ^b^ Pearson’s correlations (r). ^c^ Spearman’s correlations (Rho). ^d^ F-, *p-* and η^2^-statistics are based on logarithmized AUC_G_ scores, M and SD are shown as non-logarithmized values.

**Table 3:** Stress reactivity and depressive symptoms severity before and after six weeks of aerobic exercise training.

		Baseline		Post-Intervention		Time			Group			Time × Group Interaction		
Stress reactivityas assessed via …	*n*	M	SD	M	SD	F	*p*	η^2^	F	*p*	η^2^	F	*p*	η^2^
														
AUC_G_^a^ in the …						0.1	0.798	0.003	1.7	0.209	0.068	0.0	0.860	0.001
Total sample	25	452	265	441	242									
Intervention group	14	423	301	393	215									
Control group	11	489	219	501	272									
														
Depressive symptoms as assessed via the …	*n*	M	SD	M	SD	F	*p*	η^2^	F	*p*	η^2^	F	*p*	η^2^
														
BDI in the …						34.8	0.000	0.602	0.6	0.429	0.027	0.0	0.927	0.000
Total sample	25	26.4	8.6	15.7	11.1									
Intervention group	14	27.6	10.0	17.1	12.5									
Control group	11	24.9	6.6	14.0	9.2									

Notes. AUC_G_ = Area under the response curve with respect to ground. BDI = Beck Depression Inventory. ^a^ F-, *p*- and η^2^-statistics are based on logarithmized AUC_G_ scores, M and SD are shown as non-logarithmized values.
